# The association between academic achievement in physical education and timing of biological maturity in adolescents

**DOI:** 10.1371/journal.pone.0265718

**Published:** 2022-03-18

**Authors:** Ruben Vist Hagen, Monika Haga, Hermundur Sigmundsson, Håvard Lorås

**Affiliations:** 1 Department of Teacher Education, Norwegian University of Science and Technology, Trondheim, Norway; 2 Department of Psychology, Norwegian University of Science and Technology, Trondheim, Norway; Qatar University College of Education, QATAR

## Abstract

Individual differences in tempo and timing of biological maturity, especially in adolescents, has been argued as a potential underlying cause of relative age effects observed in Physical education (PE). Indeed, differences in maturation could influence pupils`achievement in PE where motor behavior and physical activity are central tenets. However, the timing of biological maturity has not previously been investigated in the context of academic achievement in PE. Therefore, the current study`s aim was to investigate the association between timing of biological maturity and relative age on adolescents`academic achievement in PE. The data material consists of two samples from lower secondary schools (13–16 years old). Sample 1 (45 boys and 31 girls) was used to compare differences in anthropometrics and indicators of biological maturity between pupils with different grades (i.e., 3/4, 5, 6), while in sample 2 (114 boys and 127 girls) differences in mean grade were compared between birth quartiles. Pupils`final grade in PE were collected for both samples as a proxy for academic achievement. A one-way analysis of variance indicated a moderate-to-large association between timing of biological maturity, through age at peak height velocity, and academic achievement in PE, where later maturing pupils received a higher grade compared to earlier maturing pupils. Results from a factorial analysis of variance indicated an interaction effect between gender and birth quartile on academic achievement in PE. Post hoc analysis revealed that relatively younger boys received significantly lower grades as opposed to their relatively older peers. This effect was not present for girls. Collectively, these results indicate that developmental differences are associated with academic achievement in PE. Hence, PE teachers should be aware of these individual constraints in their teaching and assessment practice to accommodate fair and equal opportunities for achievement in the subject.

## Introduction

As a measure of academic achievement, the grade in Physical education (PE) should reflect a pupil`s attained level of knowledge and skills in accordance with the curriculum`s formulated learning outcomes [1]. Importantly, the grade in PE serves as a selection instrument in the progress towards higher education and/or employment together with academic achievement from other school subjects that are typically summated and ranked [2]. Additionally, learning outcomes described in the Norwegian PE curriculum indicate that teachers`assessment of pupils`achievement level should consider their individual prerequisites (e.g., developmental differences) [3]. Thus, it is of importance that academic achievement in PE reflects a fair and objective assessment procedure. However, multiple constraints influence the evaluation of academic achievement and thus represent a challenge for pupils, teachers and educators [4]. The traditional grouping of pupils into chronological and annual age cohorts, is one example of schools trying to level out some of the developmental differences among pupils in delivery of teaching content and assessment [5]. Nevertheless, this results in a potential 12-month difference between pupils within the same annual age group cohort. Consequently, academic achievement can still potentially be confounded by developmental differences between pupils [6].

Biological maturity and relative age represent developmental differences with the potential of influencing pupils`achievement in PE [5,7]. Viewed in the perspective of the constraints-led framework, these developmental differences are considered as individual constraints where their dynamic interaction with environmental (e.g. the school) and task (i.e. PE content) constraints act upon teachers`assessment procedures [8]. Biological maturation is considered a process towards the mature state and is characterized by diversity in tempo and timing, where tempo describes the rate of maturation within a given biological system, and timing is a reference to specific maturational events in relation to age (e.g., onset of puberty) [9]. During the adolescent growth spurt the age at peak height velocity (APHV) represent a somatic indicator of biological maturity timing, associated with the age at maximum rate of growth in height [10]. On average, girls reach peak height velocity at around 12 years of age and boys around 14 years of age [11]. However, there are considerable variations in APHV among individuals of the same chronological age with ranges of 9.3–15.0 and 11.9–16.72 reported for British, Swiss and Polish girls and boys, respectively [10,12,13]. As timing of biological maturity may have meaningful consequences for biopsychosocial development [14], any research investigating academic achievement during lower secondary school (13–16 years) should acknowledge biological maturity as a potential influential constraint.

During adolescence, growth and maturational processes are associated with the development of several physical attributes such as greater height and weight, and improved strength, speed and endurance [10]. As motor behavior and physical activity are central tenets of PE, it is potentially the subject where maturational attributes are most prominent compared to other academic subjects. Physical education is also a subject where traditional forms of assessment have been argued to be more product–or performance oriented through the application of physical fitness tests and focus on sport-related skills [15–19]. In the context of sport performance, the more maturational advanced adolescents seem to have an advantage compared to their less advanced peers in a variety of sport activities, indicating that timing of biological maturity is strongly associated with performance [20–22]. As it is argued that PE is dominated by a sport and exercise discourse and activities [23–26], important performance indicators such as maturity, with concomitant development of physical attributes, can contribute to advantages and disadvantages in relation to pupils`academic achievement in PE. In particular, this may be relevant in lower secondary school, as individual differences in both physical attributes and physical performance can be heightened during the adolescent growth spurt [20,27].

Relative age describes the potential 12-month age difference between pupils within the same chronological age group cohort, while resultant consequences of this age difference are described as relative age effects (RAE) [6]. There is growing evidence for this effect upon academic achievement in PE. Relatively older pupils receive a higher grade compared to their relatively younger peers in both the UK (pupils of 11–14 years old) [5,28,29], and in Norway (13–16 years old and 16–18 years old) [7,30]. In addition, Bell et al. [31] found ratings of sport performance to be affected by relative age in 16-year-olds taking a General Certificate of Secondary Education in PE. Almost without exception, RAEs have been present irrespective of gender. Interestingly, findings in Aune et al. [30] indicated a greater magnitude of RAE for girls compared to boys in lower secondary school (15–16 years), while there was no effect of relative age for girls attending their last year of upper secondary school (18–19 years) in PE. It is suggested that earlier maturing girls might have a disadvantage over later maturing girls due to increased relative fat mass and decreased relative muscle mass [10]. This could also explain the lack of RAEs in upper secondary school for girls as it represents a point in development where most girls would have gone through puberty and consequently any maturational related advantages or disadvantages could be reduced.

Physiological growth and maturation have been argued as one of the main causes behind RAE [6,32], suggesting that there is a relationship between relative age and maturity. However, in the sport domain, relative younger individuals may counter the RAE if they are more maturational advanced [21,33], while there is substantial variability in effect sizes for RAEs observed in PE, from small to large [7,28–30]. Additionally, RAEs are previously found for numeracy and reading literacy [34,35], where maturity associated physical advantages should be of no importance. This highlights the independent nature of these two constructs, with separate factors underlying biological maturity and relative age. The former being a result of both genetic and environmental factors [10], while the latter a result of birth date in relation to schools`cutoff dates. In Norwegian schools, pupils born in a certain year between the cutoff dates 1^st^ of January and 31^st^ of December belong to the same age cohort, while September 1^st^ acts as the cutoff point in the UK education system. As a result, it is not necessarily RAE in itself, but rather timing of maturity with its accompanied advanced physical attributes that is associated with academic achievement in PE.

To the authors`knowledge, there is only one previous study addressing the potential role of physical growth (i.e., height and weight) in relation to grading in PE. Dalen et al. [7] found high achieving pupils to be significantly taller compared to lower achieving pupils. The study population`s age (13–16 years) is associated with the adolescent growth spurt, where individual differences in timing and tempo of maturation could be considered to be at its greatest [10]. However, height and weight alone could be considered a gross proxy [36], as it alone does not account for intra-individual variation in timing and tempo of growth between leg length and upper body [37]. Furthermore, it does not account for gender specific differences in maturation. Thus, there is a need to further investigate the association between maturational differences and academic achievement in PE.

Based on the presented considerations, which highlighted a sport and activity dominated PE-subject, and considerable individual developmental differences among adolescents, the aim of the current study was to investigate the association between timing of biological maturity and relative age on academic achievement in physical education for pupils attending lower secondary school (13–16 years). In order to provide an indication of biological maturity timing, a non-invasive prediction equation of biological maturity was used [37]. It was hypothesized that 1) earlier maturing pupils would receive higher grades compared to later maturing pupils, and 2) relatively older pupils would receive higher grades compared to their relatively younger peers for both boys and girls.

## Materials and methods

### Participants

Data on maturity and relative age was collected through two different data collections, and therefore two samples on Norwegian pupils are included in this study. One for the investigation of biological maturity timing (N = 76) and one for RAE (N = 241) in relation to the grade received in PE. Only public schools, being coeducational and following the same curricula, where invited for participation. Additionally, pupils were included in the study if they followed normal school progression according to their year of birth and obtained a grade in PE.

#### Sample 1 –anthropometry and timing of biological maturity

Forty-five boys and 31 girls (N = 76) from 8^th^-10^th^ grade in lower secondary school participated in this study, providing data on anthropometrics, biological maturity timing and grade. These pupils had a mean age of 14.43 (SD = .76) when data for anthropometrics and timing of biological maturity was gathered during the spring of 2020. Average grade for this sample was 5.02 (SD = .72) for boys and 4.81 (SD = .48) for girls. This sample represents one of the two schools included in sample 2. From sample 1, 41 pupils had not participated in the previous data collection during the spring of 2019, and therefore were included in sample 2 as well.

#### Sample 2 –relative age

One hundred and fourteen boys and 127 girls (N = 241) from 8^th^-10^th^ grade in lower secondary school participated in this study, providing data on relative age and grade in PE. The pupils had a mean age of 14.46 (SD = .70) when end of year grade was obtained. Data was gathered during the spring of 2019 for 200 of these pupils, while the remaining 41 pupils was included from sample 1 (see above). The sample represents two schools varying in location and size, from city to suburb, with the aim to include pupils with different sociocultural- and economic backgrounds. Average grade for this sample was 4.88 (SD = .74) for boys and 4.59 (SD = .82) for girls.

### Ethical considerations

All pupils and their legal guardians signed a written informed consent in advance of data collection. It was made clear that participation was voluntary, that they had the option to withdraw at any time, and that their data would not be disclosed to anyone except the project leader. The Norwegian Centre for Research Data (NSD) approved the study protocol (#169464).

### Procedure and measurements

#### Grade

As a proxy for academic achievement, the participants’ grade in PE was collected in the month of June 2020 for sample 1 and June 2019 for sample 2. For sample 1, this was approximately 3 months after collecting anthropometric data. Pupils`grade and related data were linked by distinctive ID numbers allocated to each pupil. In the Norwegian school system, grades are scored on a scale from 1–6, where 6 is the highest grade [1]. The national average grade in 10^th^ grade was 4.7 for boys and 4.6 for girls in 2019 and 4.7 for both boys and girls in 2020 [38,39]. Thus, the average grade for both samples was quite similar to the national average.

#### Anthropometry and timing of biological maturity

Following previous procedures [12,40], height and sitting height (Seca 213 Portable Stadiometer, Hamburg, Germany) were measured to the nearest 0.1cm. Leg length was calculated as the difference between height and sitting height. Body weight (Seca 803 Electronic Flat Scale, Hamburg, Germany) was measured to the nearest 0.1kg. All measurements were taken barefoot, wearing shorts and a t-shirt. Two measurements were taken for each anthropometric variable, and an average value calculated. If the two measurements differed by more than 0.4cm for height and sitting height, and 0.4kg for weight, a third measure was taken, using the median value [37].

Including the abovementioned anthropometric measurements is the non-invasive prediction equation of biological maturity [37]:

$$\left(\text{Boys}\right):\;\text{Maturity}\;\text{offset}\;\left(\text{years}\right)=-9.236+(\left(0.0002708\;\text{x}\;\left(\text{leg}\;\text{length}\;\text{x}\;\text{sitting}\;\text{height}\right)\right)+\left(-0.001663\;\text{x}\;\left(\text{age}\;\text{x}\;\text{leg}\;\text{length}\right)\right)+\text{(}0.007216\;\text{x}\;\left(\text{age}\;\text{x}\;\text{sitting}\;\text{height}\right)+\left(0.02292\;\text{x}\;\left(\text{mass}\;\text{by}\;\text{stature}\;\text{ratio}\;\text{x}\;100\right)\right).$$ Eq 1

$$\left(\text{Girls}\right):\;\text{Maturity}\;\text{offset}\;\left(\text{years}\right)=-9.376+\left(0.0001882\;\text{x}\;\left(\text{leg}\;\text{length}\;\text{x}\;\text{sitting}\;\text{height}\right)\right)+\left(0.0022\;\text{x}\;\left(\text{age}\;\text{x}\;\text{leg}\;\text{length}\right)\right)+\left(0.005841\;\text{x}\;\left(\text{age}\;\text{x}\;\text{sitting}\;\text{height}\right)\right)–\left(0.002658\;\text{x}\;\left(\text{age}\;\text{x}\;\text{mass}\right)\right)+\left(0.07693\;\text{x}\;\left(\text{mass}\;\text{by}\;\text{stature}\;\text{ratio}\;\text{x}\;100\right)\right).$$ Eq 2

The maturity offset represents predicted years from peak height velocity, with a negative value indicating that the pupil has still to reach peak height velocity and a positive value indicating that it has passed. The APHV is calculated as the difference between chronological age and maturity offset.

#### Relative age

The allocation of Norwegian pupils into grades range from 1^st^ January– 31^st^ December. By using the cutoff date and pupils`birth date, relative age was established. Further, according to birth date, pupils were categorized into four quartiles (Q1: January-March; Q2: April-June; Q3: July-September; Q4: October-December). Pupils born in Q1 are the relatively oldest.

### Statistical analysis

Statistical analysis was performed using IBM SPSS version 25.0. Prior to main analysis, data was tested for normality, skewness and kurtosis. For sample 1, the variables age and weight was transformed towards normality through a two-step approach [41]. This retains the original series mean and standard deviation, which helps interpretation of results.

For sample 1, a one-way analysis of variance (ANOVA) was used to address any gender specific differences in grade, anthropometric measurements (i.e. weight, height, sitting height and leg length) and indicators of biological maturity timing (i.e. maturity offset and APHV). For the first hypothesis, a one-way ANOVA compared the mean value of age, anthropometric measurements and indicators of biological maturity timing between groups of grade (i.e. 3/4, 5 and 6) for the total sample 1. As there were no significant differences in grade between boys and girls in this sample, we did not further address gender specific differences in the previously mentioned variables in relation to the grade. For the second hypothesis, the effect of relative age was examined with a 2 (gender) X 3 (year of study) X 4 (birth quartile) factorial ANOVA on grade in PE for sample 2. Grade may be affected by gender, in favor of boys, and time spent in lower secondary school/year of study (i.e., grades 8 vs. 9 vs. 10) [28,42]. Hence, potential main effects, interactional effects and simple effects were of relevance in this study. Where necessary, post hoc Least Significant Differences corrected pairwise comparisons were used to address potential differences between groups. Effect sizes were reported as partial eta squared (η2) and interpreted as small (0.01), medium (0.06) and large (0.14) [43,44]. Statistical significance level was set at p < .05.

## Results

### Anthropometry, timing of biological maturity and academic achievement in PE

Table 1 outlines the descriptive statistics (M ± SD) for grade, anthropometric measurements and indicators of biological maturity timing by gender. Boys were significantly taller in standing (F(1,74) = 20.30, p = .000; η2 = .215) and sitting height (F(1,74) = 6.69, p = .012; η2 = .083) compared to girls. Boys had also significantly longer legs (F(1,74) = 28.98, p = .000; η2 = .281), and were significantly heavier than the girls (F(1,74) = 5.34, p = .024; η2 = .067). Further, girls had a significantly greater maturity offset compared to boys (F(1,74) = 45.80, p = .000; η2 = .382), and reached their APHV significantly earlier than boys (F(1,74) = 104.50, p = .000; η2 = .585). The sample`s average APHV for girls and boys were in line with the expected average value of 12 and 14 years, respectively [10]. There were no significant differences in mean grade between boys and girls (F(1,74) = 2.12, p = .150; η2 = .028).

**Table 1:** Grade, Anthropometrics and indicators of biological maturity timing by gender (N = 76).

Variables	Girls (N = 31) (M/SD)	Boys (N = 45) (M/SD)	Total (N = 76) (M/SD)
**Grade**	4.81 ± .48	5.02 ± .72	4.93 ± .64
Weight (kg)*	54.12 ± 7.42 (54.64 ± 10.51)	60.85 ± 14.67 (61.54 ± 14.14)	58.11 ± 12.63 (58.72 ± 13.16)
Height (cm)*	163.92 ± 6.49	171.52 ± 7.69	168.42 ± 8.11
Sitting height (cm)*	85.06 ± 2.90	87.62 ± 4.93	86.57 ± 4.39
Leg length (cm)*	78.86 ± 4.48	83.91 ± 3.68	81.85 ± 4.71
Maturity offset*	2.04 ± .67	.70 ± .94	1.24 ± 1.06
APHV*	12.39 ± .43	13.75 ± .65	13.19 ± .88

SD = Standard Deviation; M = Mean; APHV = Age at Peak Height Velocity. * Significant difference between gender (p < .05). Note. Values in parenthesis are mean and SD after transformation. For variables with parenthesis, all were transformed by the two step process.

Table 2 outlines the descriptive statistics (M ± SD) for age, anthropometric measurements and indicators of biological maturity timing for each group of grades. None of the anthropometric variables height (F(2,73) = .16, p = .854; η2 = .004), sitting height (F(2,73) = .63, p = .536; η2 = .017), leg length (F(2,73) = .15, p = .863; η2 = .004) or weight (F(2,73) = 1.48, p = .236; η2 = .039) differed significantly between grades. Neither did chronological age (F(2,73) = .87, p = .423; η2 = .023) or maturity offset (F(2,73) = .94, p = .396; η2 = .025). However, APHV differed significantly between grades (F(2,73) = 3.60, p = .032; η2 = .090), where a pairwise comparison revealed that pupils receiving a grade of 6 reach APHV significantly later compared to pupils with a grade of 5 (mean difference = .703, 95% CI (.156: 1.251)) and 3/4 (mean difference = .756, 95% CI (.108: 1.404)). For those pupils receiving a grade of 6, the mean and standard deviations for maturity offset indicated that some of these pupils had still to reach their APHV (Table 2).

**Table 2:** Age, anthropometrics and indicators of biological maturity timing by grade (N = 76).

Variables/grades	3/4 (M/SD)	5 (M/SD)	6 (M/SD)	Total (M/SD)
**Age (y)**	14.46 ± .79 (14.46 ± .79)	14.36 ± .69 (14.40 ± .75)	14.67 ± 1.00 (14.74 ± 1.00)	14.43 ± .76 (14.46 ± .80)
**Weight (kg)**	63.33 ± 19.29 (63.50 ± 17.24)	56.50 ± 10.62 (57.03 ± 12.29)	57.57 ± 6.84 (59.11 ± 9.15)	58.11 ± 12.63 (58.72 ± 13.16)
**Height (cm)**	168.63 ± 10.22	168.07 ± 7.78	169.53 ± 6.74	168.42 ± 8.11
**Sitting height (cm)**	87.33 ± 5.33	86.14 ± 4.00	87.30 ± 4.68	86.57 ± 4.39
**Leg length (cm)**	81.31 ± 6.12	81.93 ± 4.53	82.23 ± 3.44	81.85 ± 4.71
**Maturity offset**	1.42 ± 1.06	1.26 ± 1.05	.88 ± 1.15	1.24 ± 1.06
APHV*	13.04 ± 1.10	13.09 ± .84	13.79 ± .43	13.19 ± .88

SD = Standard Deviation; M = Mean; APHV = Age at Peak Height Velocity. *Significant difference between groups of grade by ANOVA (p < .05). Note. Values in parenthesis are mean and SD after transformation. For variables with parenthesis, all were transformed by the two step process.

### Relative age and academic achievement in PE

The 2 X 3 X 4 ANOVA indicated a significant overall effect of the model on grade in PE (F(23,217) = 1.794, p = .017; η2 = .160). The effect of gender X year of study X birth quartile interaction was not statistically significant (F(6,217) = 1.158, p = .330; η2 = .031). Neither were there any interaction effects for gender X year of study (F(2,217) = .693, p = .501; η2 = .006) or year of study X birth quartile (F(6,217) = .325, p = .923; η2 = .009). However, there was a significant gender X birth quartile interaction effect on grade in PE (F(3,217) = 3.435, p = .018; η2 = .045). Further, the ANOVA indicated a significant main effect of gender (boys vs. girls) on grade in PE (F(1,217) = 4.639, P = .032; η2 = .023), and for year of study (grades 8 vs. 9 vs. 10) (F(2,217) = 5.003, p = .008; η2 = .044). No significant main effect was evident for birth quartile (Q1 vs. Q2 vs. Q3 vs. Q4) (F(3,217) = .773, p = .510; η2 = .011).

**Fig 1:**
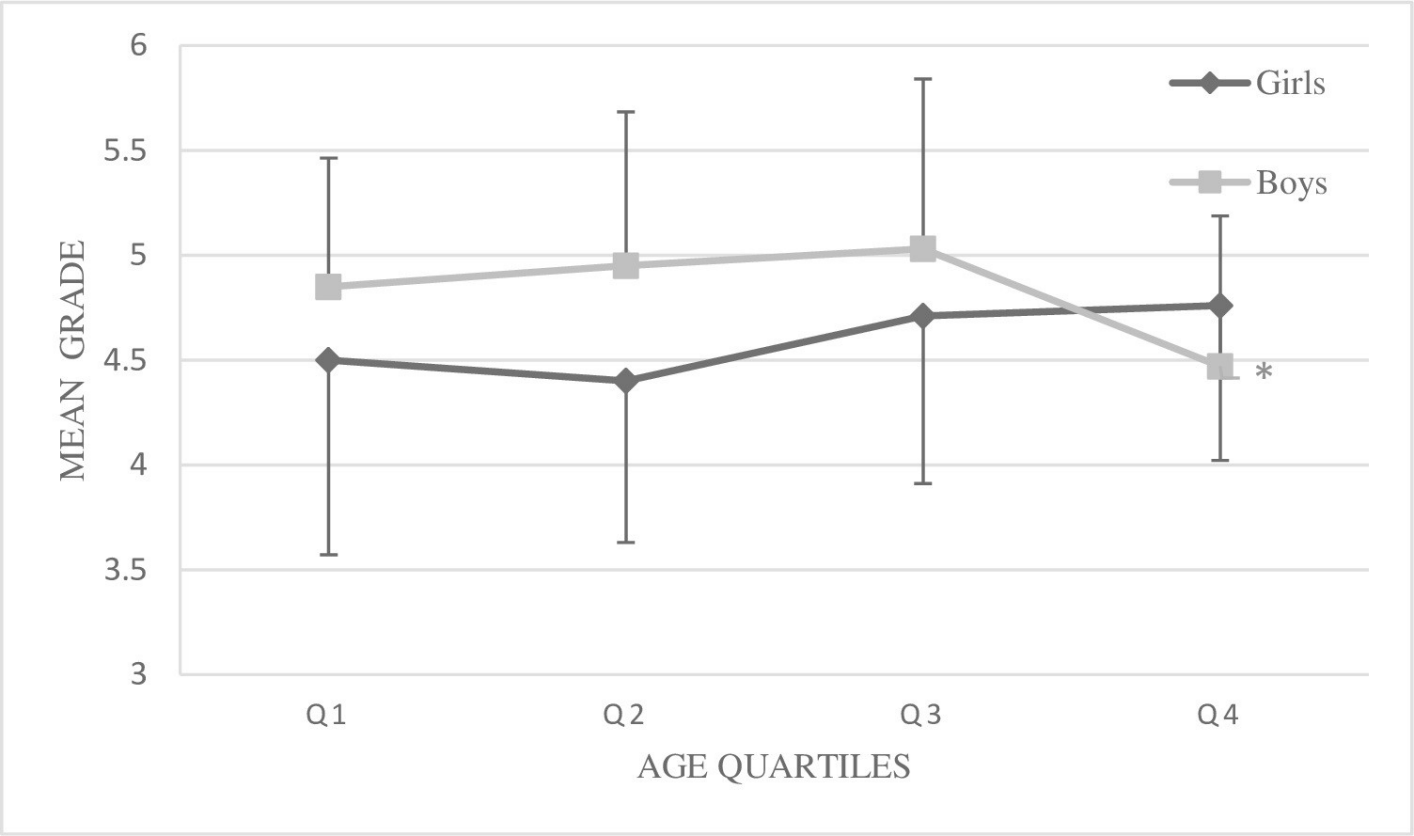
Mean grade and SD for boys and girls across birth quartiles (N = 241). *Significantly lower mean grades for pupils born in the 4^th^ quartile compared to pupils born in the 2^nd^ and 3^rd^ quartile.

Given the significant interaction effect of gender X birth quartile, simple effects were calculated and pairwise comparisons with a least significant difference correction was applied. Only for Q2 did boys have a higher mean grade compared to girls with a mean difference of .547 (p = .004, 95% CI (.147: .921)) (Fig 1). Effect of birth quartile was significant in the pairwise comparisons for boys only, where those born in Q2 (mean difference = .477, p = .036, 95% CI (.030: .923)) and Q3 (mean difference = .560, p = .017, 95% CI (.103: .1.017)) received higher grades compared to Q4 (Fig 1). Main effects of gender and year of study were also further explored with a least significant difference correction to the pairwise comparisons. There was a mean difference in grade of .358 (p = . 032, 95% CI (.030; .686)) in favor of boys, and a mean difference of .343 (p = .003, 95% CI (.117: .569)) between 8^th^ and 9^th^ grade in favor of the latter.

## Discussion

The main aim of his study was to investigate the association between the timing of biological maturity and relative age on academic achievement in PE in adolescents. The main findings indicated a moderate-to-large association between APHV and academic achievement in PE, in which the later maturing pupils received a higher grade as opposed to earlier maturing pupils. Furthermore, a moderate-to-small interaction effect between gender and birth quartile was found, indicating relative age effect among boys, in which relatively older pupils had a higher academic achievement in PE compared to their relatively younger peers.

### Anthropometry, timing of biological maturity and academic achievement in PE

The current sample`s APHV was in line with the average values for both boys (14 years) and girls (12 years) [11]. Furthermore, the boys were on average both taller and heavier compared to girls, which is expected for this age group [10]. However, this did not result in any significant difference in academic achievement in PE between the boys and girls, which might be reasonable given that the current samples`average achievement corresponds with nation-wide average PE academic achievement in lower secondary school [39]. Due to no statistically significant gender differences in achievement, no further gender specific differences in APHV were examined in subsequent analysis.

As opposed to our hypothesis of earlier maturing pupils receiving higher grades, the current study`s results indicate a novel finding in that the less maturational advanced pupils obtained the highest achievement. Pupils receiving the highest grade had a significant later predicted APHV compared to pupils obtaining lower achievement. This finding is in contrast to previous research in both the sport domain, particularly team sports, and PE, which indicated that individual constraints such as more advanced maturational- or growth characteristics resulted in performance- or achievement advantages [7,22,45]. For instance, earlier maturing adolescents participating in selection camps seemed to have a greater chance for being selected to an elite hockey team as opposed to later maturing adolescents [22]. In a PE-context, pupils with more advanced growth characteristics (i.e. taller individuals) has been found to have higher academic achievement in PE [7]. A possible (although speculative) explanation for the current study`s finding might be the suggested period of instability, regression or delay in motor coordination in the immediate period after the adolescent growth spurt [46–48]. Consequently, the more mature pupils in our study may have experienced a temporary variability in motor competence/coordinative ability, affecting their motor skill level and in turn their achievement in PE. Support for this can be found in a longitudinal study on Canadian youth, in which Sheehan and Lienhard [49] observed a reduction in physical qualities for boys and girls after experiencing their growth spurt, before initially catching up in the subsequent years. More specifically, boys experienced a reduction in performance for running speed and agility, while girls had a reduction in strength, agility and gross motor skills. These could all be considered important individual constraints in PE due to the sport- and fitness inspired teaching content as well as the traditional use of physical fitness tests and focus on isolated motor skills in assessment [15,16,18,26]. However, maturation may have a limited impact on motor coordination, as skeletal maturity status have been found to explain only 8.1% and 2.8% of the variance in motor coordination among 11–14 year old boys and girls, respectively [50].

In the current study, neither of the anthropometric variables explained differences in PE academic achievement. Dalen et al. [7] previously reported that taller pupils received higher academic achievement in PE and argued that this is potentially related to earlier physical maturation accompanied by physical fitness advantages such as improved strength, endurance and speed. However, as pupils with different academic achievement differed in their APHV, this study is to some degree confirming the previous finding of a statistical association between biological maturation and the academic achievement of pupils in PE.

A potential rationale for the lack of height alone having a statistical effect on academic achievement in our study, could be due to the fact that there are substantial gender specific and individual differences in the rate and timing of growth between body parts [37], which is accounted for when assessing predicted APHV. It should also be noted that Dalen et al. [7] assessed a considerably larger sample size compared to our study. Reasonably, they were perhaps able to detect a more accurate or meaningful effect of height on academic achievement in PE, while the variability in our smaller sample size could obscure a meaningful difference. However, chronological age was not associated with academic achievement in the current study, suggesting an independent relationship between chronological age and maturity where timing and tempo varies greatly through the adolescent growth spurt.

### Relative age and academic achievement in PE

The current study further corroborates previous studies highlighting the systematic bias of birth date acting as an individual constraint on academic achievement in PE [5,7,28–31]. The interaction effect of gender by birth quartile revealed that RAE was present for boys, where the relatively older received a higher academic achievement compared to their relatively younger peers. It should be noted that this interaction effect is interpreted as small to moderate, and that only boys in quartile 2 and 3 got a statistically significant better academic achievement than those born in the last quartile. The mean academic achievement for quartile 1 was higher compared to quartile 4, however not statistically significant. These results portray a much less clear or obvious RAE as opposed to a stronger negative linear correlation between birth quartile and academic achievement seen in previous studies [5,7,28–30].

There are, however, more conflicting results concerning RAE for girls in PE. In accordance with our sample, Dalen et al. [7] found no RAE for girls in a similar population of Norwegian pupils in lower secondary school. As girls reach their APHV around 12 years of age, the age group (13–16 years) for these samples could suggest that most of the girls had passed their growth spurt. Consequently, any possible advantages related to maturity that could be associated with RAE would have been reduced or diminished all together. This could also explain why Roberts and Fairclough [29] found RAE for 11–14 year old girls, potentially representing an age span including girls who have yet to reach their growth spurt, resulting in the potential for maturational advantages or disadvantages to have been present. Aune et al. [30] however, found the magnitude of RAE in PE academic achievement to be greater for girls than boys in a similar age group as the participants in the current study. These conflicting findings point towards two important implications. Firstly, due to the great individual variations in timing and tempo of maturation, the presence of RAE could be dependent upon the degree of individual differences in the sample studied. Secondly, it might provide an indication for the independent nature between RAE and maturity as pointed out in the introduction, and that there might instead be other mechanisms influencing the RAE to a greater degree.

Another important finding to consider, leaning towards a moderate effect, is the role of time spent (experience) in PE (i.e., year of study) as in the current study a significant difference in academic achievement between 8^th^ and 9^th^ grade was found, in favor of the latter. Similar findings have been reported elsewhere [5,28]. With quantity and quality of practice being some of the important explanatory mechanisms of performance [51,52], level of academic achievement in PE could rather be a result of experiences or time accumulated in PE throughout the years spent in school. Additionally, due to the sport inspired teaching content in PE [26], participation in recreational or sport activities outside of school may be of additional interest for future research as it may provide specific carry-over effects into activities in PE. It should be noted that the Norwegian curriculum`s learning outcomes are expected to be reached by 10^th^ grade. Even though pupils receive a grade in PE prior to graduating 10^th^ grade, these do not represent their final level of achievement. Therefore, pupils`work in relation to these learning outcomes should be progressed through difficulty and complexity of teaching content from 8^th^ to 10^th^ grade. In this sense, assessment could be considered ipsative and may act as a logical explanation for the progressively higher academic achievement seen between grades.

### Practical implication

Regardless of either less or more advanced maturational characteristics influence academic achievement in PE, the presence of such an effect, as well as RAE, could be considered as a bias in the PE teachers`assessment practice. Especially when the formulated learning outcomes in the Norwegian PE curriculum enables teachers to account for pupils`individual prerequisites in their assessment practice [3]. Potentially, the maturity associated development of physical abilities such as speed, strength and endurance may give significance to pupils`timing of biological maturity due to the sport-oriented activity focus as well as the use of more performance -oriented assessment strategies. Based on the current study`s findings, it may be reasonable to encourage a greater variety in teaching content and methodology used in PE, as well as reconsidering the use of performance-oriented assessment to better provide equal opportunities for learning and achievement in PE classes, as individual differences may vary greatly.

### Methodological considerations

Due to addressing timing of biological maturity and relative age in different samples we could not directly explore these variables`relationship–whether they are two independent constructs or if timing of biological maturity is a moderator of RAE in PE, which is an important avenue for further research. Also, both samples in this study had a high average academic achievement in PE, however, in proximity with the national average for 10^th^ grade, suggesting that the samples contained some resemblance to the general population in the Norwegian lower secondary school. Prior to data collection measures were taken to ensure we recruited as broadly as possible. This consisted of informing both pupils and legal guardians about the process of the data collections as well as the purpose of the research. For sample 1, we specifically planned for the assessment of anthropometric variables to be conducted in a safe and private environment in order to reduce the influence of a selection bias, where pupils who potentially are more confident in their skills/appearance choose to participate. Finally, the predictive equation of biological maturity timing can be considered a practical measurement tool being time-efficient, relatively non-invasive, and reliable. However, it should be appreciated that the calculated APHV from this equation is often lower than actual APHV for individuals who has yet to reach their growth spurt and higher for individuals who have passed it [12]. Consequently, one can underestimate and/or overestimate pupils biological maturity timing.

## Conclusions

Results of the current study indicated that timing of biological maturity and relative age are associated with academic achievement in PE among adolescents. In contrast to the hypothesis of earlier maturing pupils receiving a higher academic achievement, there was a significant difference in APHV between pupils where less maturational advanced pupils received the top academic achievement. Regarding RAE, the results confirmed the hypothesis of relatively older pupils receiving higher academic achievement in PE compared to their relatively younger peers. However, this was evident for boys only. Collectively, these results offer new insight into individual constraints associated with academic achievement in PE, which is of importance to account for in order to provide fair assessment of pupils in PE. More specifically, PE-teachers should acknowledge the individual differences in timing and tempo of biological maturation and the associated physical advantages in their teaching and assessment practice, as well as look for systematic bias in their assessment considering relative age. In line with the constraints-led framework, a potential avenue for future studies is to investigate the dynamic and mutually influential relationship between environmental, task and individual constraints to better understand the complex nature of pupils`academic achievement in PE.
